# Vitronectin as a molecular player of the tumor microenvironment in neuroblastoma

**DOI:** 10.1186/s12885-019-5693-2

**Published:** 2019-05-22

**Authors:** Rebeca Burgos-Panadero, Inmaculada Noguera, Adela Cañete, Samuel Navarro, Rosa Noguera

**Affiliations:** 1 Pathology Department, Medical School, University of Valencia-INCLIVA, Valencia, Spain; 2 CIBERONC, Madrid, Spain; 3 Central Support Service for Experimental Research (SCSIE), University of Valencia, Valencia, Spain; 4 Pediatric Oncology Unit, University and Polytechnic Hospital La Fe, Valencia, Spain

**Keywords:** Extracellular matrix, Vitronectin, Digital pathology, Migration, Neuroblastoma

## Abstract

**Background:**

Vitronectin is a multifunctional glycoprotein known in several human tumors for its adhesive role in processes such as cell growth, angiogenesis and metastasis. In this study, we examined vitronectin expression in neuroblastoma to investigate whether this molecule takes part in cell-cell or cell-extracellular matrix interactions that may confer mechanical properties to promote tumor aggressiveness.

**Methods:**

We used immunohistochemistry and image analysis tools to characterize vitronectin expression and to test its prognostic value in 91 neuroblastoma patients. To better understand the effect of vitronectin, we studied its in vitro expression using commercial neuroblastoma cell lines and in vivo using intra-adrenal gland xenograft models by immunohistochemistry.

**Results:**

Digital image analysis allowed us to associate vitronectin staining intensity and location discriminating between territorial vitronectin and interterritorial vitronectin expression patterns. High territorial vitronectin expression (strong staining associated with pericellular and intracellular location) was present in tumors from patients with metastatic undifferentiating neuroblastoma, that were *MYCN* amplified, 11q deleted or with segmental chromosomal profiles, in the high-risk stratification group and with high genetic instability. In vitro studies confirmed that vitronectin is expressed in tumor cells as small cytoplasmic dot drops. In vivo experiments revealed tumor cells with high and dense cytoplasmic vitronectin expression.

**Conclusions:**

These findings highlight the relevance of vitronectin in neuroblastoma tumor biology and suggest its potential as a future therapeutic target in neuroblastoma.

**Electronic supplementary material:**

The online version of this article (10.1186/s12885-019-5693-2) contains supplementary material, which is available to authorized users.

## Background

The composition, morphology and organization of the extracellular matrix (ECM) is key to both healthy and pathological environments. In healthy tissue, the ECM regulates development and homeostasis, whereas in tumors it displays mechanical properties such as stiffness that confer malignant characteristics to cell behaviour including proliferation, cell-death resistance, angiogenesis, invasion and metastasis [1–3]. The interaction between tumor cells and their surrounding elements is the first step in the development of metastasis, since cell movement requires firm cell-ECM adhesions to break down, as well as molecules to guide the migration. Different cancer-promoting biological pathways of interest for further exploration are cell-cell or cell-ECM adhesions, proteases and chemokines [4]. In addition, recent studies show that cancer invasion and metastasis are driven by physical and chemical interactions between tumor cells and the ECM that translate into a stiff neoplastic ECM and soft or deregulated tumor cells, which in turn lead to a more favorable microenvironment for cancer dissemination [5–8].

Vitronectin (VN) is an adhesive glycoprotein that acts as a link between cells and the ECM through several ligands such as: integrins, plasminogen activator inhibitor-1(PAI-1) and urokinase plasminogen activator receptor (uPAR). VN is present in plasma as a monomeric or dimeric structure (folded or native form) and in the ECM of several tissues as a multimeric formation (unfolded or active form) [9, 10]. It is mainly synthesized by hepatocytes in the liver, although it has also been found in smaller amounts in extrahepatic tissues such as: brain, lung, kidney and vascular wall of adrenal gland [11–13]. It has also been observed that some tumor cells secrete VN as well as tumor-infiltrating T-lymphocytes (TIL) which bind to VN through TIL uPAR expression [14, 15]. The biological functions of VN, derived from its domains which bind several ligands in its activated form, are: preservation of vascular homeostasis (thrombosis and fibrinolysis), control of the innate immune system, facilitating cell adhesion and participation in migration in tissue repair and regeneration [16]. VN has a role in the provisional matrix of tumors, where it can promote cell adhesion and matrix degradation by binding to integrins, PAI-1 and uPAR [17]. In fact, in several human neoplasms, VN is associated with tumor invasion, metastasis and angiogenesis [18–20].

Neuroblastoma (NB) originates from the neural crest in the Sympathetic Nervous System and is one of the most common pediatric solid tumors [21]. Although several clinical, biological and genetic markers define the risk of progression in NB patients [22], the mechanisms that control communication between tumor cells and the ECM, and can influence aggressiveness are not yet clear. Our group has already described the aggressive pattern of a stiff ECM defined as: ECM with cross-linked and disorganized reticulin fiber networks, scant amount of collagen type I fibers and glycosaminoglycans and large and abundant irregularly-shaped and high blood vessels, associated with a poor outcome in NB patients [23–26]. Hence, to better comprehend this tumor cell-ECM communication we searched for targets within the ECM elements. Previous studies in NB have noted VN expression in ganglion cells that could suggest a differentiation role for this molecule [12] and its α_v_β_3_ integrin receptor, which is highly expressed in high-risk NB [27].

In this study, we used digital image analysis to examine the immunohistochemical expression of VN in NB to better understand the mechanical signals between neuroblasts and the ECM and their influence on tumor growth, differentiation and dissemination. In addition, we have done in vitro and in vivo experiments to assess if VN present in tumor or host microenvironments shows any modification on NB behavior since previous research showed the relationship between VN and metastasis and tumor progression in several human neoplasms.

## Methods

### Patient samples

A total of 91 primary NB tumors (at least two representative cylinders of 1 mm) included in tissue microarrays (TMAs) were chosen according to NB genetic instability criteria [28], and classified into the following categories: very low instability (numerical chromosomal aberration (NCAs) profiles, defined as gains or losses of a whole chromosome), low instability (≤3 typical segmental chromosomal aberrations (SCAs), excluding 11q deleted (11qD) profiles, defined as gains or losses of chromosomal fragments), medium instability (profiles with *MYCN* amplified (MNA) or 11qD, both genetic markers of worse prognosis or > 3 typical SCAs) and high instability (profiles with chromothripsis, defined as a local breaking with subsequent aleatory reassembly of fragment in a single event [28], or > 3 gene amplifications), these categories were dichotomized as low instability (very low and low groups) versus high instability (medium and high groups). All samples had been referred to the Spanish Reference Centre for NB Biological and Pathological studies (Department of Pathology, University of Valencia-INCLIVA) from 2000 to 2015. The samples were also classified according to INRG clinicobiological parameters [22] (Additional file 1: Table S1). This study was approved by the Ethical Committee of the University of Valencia (reference B.0000339 29/01/2015). Participants or their family members/legal guardians provided written informed consent for histological and genetic studies performed in our laboratory. Clinical data were provided by the pediatric oncologists in charge or by the Reference center for NB clinical studies.

### Immunohistochemistry

One 3 μm section of each TMA was cut and immunostained with rabbit monoclonal antibody against VN (EP873Y, Clone; ab45139, Abcam, Cambridge, MA, USA) at 1:100 using OptiView Amplification Kit (Ventana Medical Systems Inc., Tucson, EE.UU.) in the BenchMark XT automated slide staining system (Ventana Medical Systems Inc., Tucson, USA). To determine the optimal antibody dilution, normal liver tissue and whole NB sections were used. As controls we stained several normal tissues (liver, kidney, salivary gland, smooth muscle, striated muscle, trachea, pancreas, spleen, adrenal gland, colon and placenta). Immunoreactivity was assessed by two researchers. VN immunoreaction was rated as no staining (0), and weak (1+), moderate (2+), and strong (3+). This category was dichotomized as weak to moderate vs strong. This was used to determine the adequacy of a further image analysis and help setting the image analysis parameters.

### Image analysis

All immunostained slides were digitized with the whole-slide Pannoramic MIDI scanner (3DHISTECH Ltd., Budapest, Hungary) at 20x magnification. We used two applications to quantify VN in NB samples: Image Pro-Plus (IPP) software v.6.0 (Media Cybernetics Inc., Silver Spring, MD, USA) and DensitoQuant module (DensitoQ), Pannoramic viewer software 1.15 (3DHISTECH Ltd., Budapest, Hungary). The second was used as a validation tool of the first as it allows quick segmentation based on immunohistochemical staining intensity. The steps used for IPP and DensitoQ macro customization are described in Additional file 2: Table S2. Examples of how these two applications work are provided in Additional file 3: Figure S1.

IPP: To characterize VN expression, a *macro* was customized using control tissues through RGB color segmentation; restrictive values were used to distinguish between VN staining intensity and location. We quantified VN staining as weak to moderate or strong, and VN distribution was identified as intercellular only or pericellular plus intracellular location. In addition, the percentage of VN stained area (%SA) per cylinder and the mean of the two cylinders belonging to the same case were calculated as the area positive for VN divided by the total area of the cylinder, multiplied by 100. The %SA and density (number of objects/mm^2^) of cell nuclei of each case were also quantified.

DensitoQ: The measures obtained were: negative, weak, moderate and strong pixels intensity and H-score. The H-score (or “histo” score), is a score that indicates if the sample can be considered positive or negative on the basis of a specific discriminatory threshold, ranging from 0 to 300 [29].

### Statistical methods

All data were analyzed using SPSS statistical analysis software (version 24). The consistency between the subjective assessment and the VN image analysis was analyzed using the non-parametric Kruskal-Wallis test. Samples with no immunoreactivity for VN were excluded from the statistical analysis. The VN numerical continuous variables derived from the morphometric analysis that did not follow a normal distribution were related to the INRG prognostic categories using the non-parametric Mann-Whitney and Kruskal-Wallis tests and were dichotomized using the third quartile (Q_3_) to perform a survival analysis using the Kaplan-Meier curves and log-rank test. Cox survival regression using Wald (step back) test was used to estimate the influence of VN linked to INRG prognostic factors as independent variables on event-free and overall survival (EFS and OS, respectively). We considered *p*-values less than 0.05 as statistically significant.

### In vitro and in vivo models

In vitro and in vivo models were used to evaluate the changes in neuroblasts VN expression independently of the hepatic/extrahepatic VN synthesis.

SH-SY5Y and SK-N-BE (2) NB cell lines were a generous gift from Miguel F. Segura (Laboratory of Translational Research in Child and Adolescent Cancer, Hospital Universitari Vall d’Hebron) and were grown, since VN is a fetal bovine serum component, in complete and serum-free media as indicate in Additional file 4: Table S3. To detect VN expression, cells were detached by Trypsin/EDTA 0.25% (Gibco; Thermo Fisher Scientific Inc.), deposited onto poly L-lysine coated (Sigma) slides using Shandon CytoSpin III Cytocentrifuge at 1200 rpm for 10 min, fixed with methanol/acetone (1:1) for 10 min at room temperature and stained as indicated previously.

Mice deficient in VN^−/−^ (B6.129S2 (D2)-Vtn^tm1Dgi^/J) and RAG1^−/−^ (B6.129S7-Rag1^tm1Mom^/J) were obtained from Jackson Laboratory (USA) and Charles River Laboratories (France) and interbred to homozygosis for both alleles. Four-to six-week-old female or male RAG1^−/−^ VN^+/+^ (control) and RAG1^−/−^ VN^−/−^ (experimental) mice were used for left adrenal injection of 1 × 10^6^ SH-SY5Y (*n* = 20) and SK-N-BE (2) (*n* = 20) NB cells lines in 30 μl of (1:1) Dulbecco’s phosphate-buffered saline (DPBS; Gibco; Thermo Fisher Scientific Inc.) and Matrigel (Corning; Cultek S.L.U, Barcelona, Spain). Mice were anaesthetised using 4–5% isoflurane in a glass chamber for induction and anesthetic plan was maintained by face mask with 1–2% isoflurane and subsequently buprenorphine (0.1 mg/kg) was administered subcutaneously as analgesia. All experiments were carried out in accordance with the standards and care approved by the institutional ethical animal care committee (reference 2015/VSC/PEA/00083). Tumor growth was checked visually weekly and mice were sacrificed by overdose of isoflurane at 8 weeks after taking blood from anaesthetised mice (as described previously) via the cardiac puncture method. After fixing in formaldehyde 4% and embedding in paraffin, xenograft tumors and mice organ samples were stained with Hematoxylin-eosin (HE) and with anti-VN as indicated previously. The Discovery anti-rabbit HQ reagent (Ventana Medical Systems Inc., Tucson, EE.UU.) was only used to detect VN expression in human NB cells.

## Results

### VN is present in NB samples

**Fig. 1:**
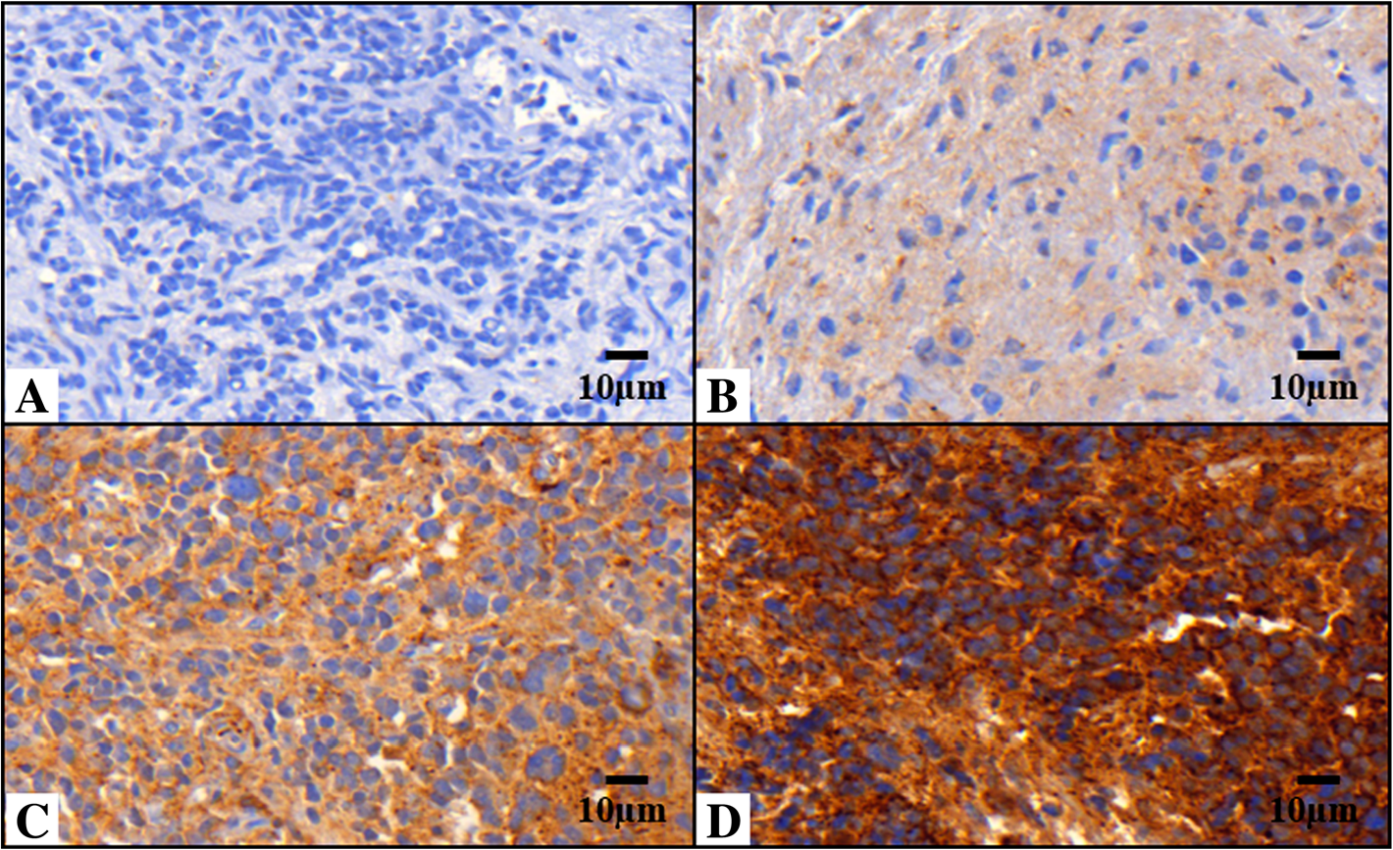
Vitronectin pattern in neuroblastic tumors. Images immunostained with antibody anti-vitronectin (VN) at 40X. **a** Sample corresponding to negative VN. **b** and **c** Samples corresponding to weak to moderate VN expression and ECM distribution only (defined as interterritorial VN). **d** Sample with strong VN expression with pericellular and intracellular location (defined as territorial VN)

Positive VN immunoreactivity was observed in 85 out of 91 samples (93.4%). A good consistency between subjective and digital image analysis was observed (*p*-value = 0.000). The subjective assessment of VN pattern was weak to moderate (61 samples) and strong, (24 samples) (Fig. 1). Using digital image analysis, we found that: 1) Strong VN intensity was associated with a pericellular and intracellular location; it is mostly present in the pericellular region and stored intracellularly to a lesser extent. The pericellular region concerned two tiny sites: the matrix adjacent to the cell membrane like a capsular territory, and a layer surrounding the capsular matrix of each cell or nests of grouped cells; we labeled this expression pattern territorial VN. 2) Weak to moderate VN intensity was associated with intercellular location (peripherally to territorial matrix); we named this expression interterritorial VN (Fig. 1).

The objective quantification patterns were considered as the %SA of interterritorial VN, territorial VN and H-score. We noted that the %SA of interterritorial VN and H-score was low in control tissues, high in liver tissues and intermediate in NB samples. Nevertheless, the highest %SA of territorial VN was found in NB samples. All VN and nuclei morphometric measurements are shown in Table 1. Median values of VN and nuclei quantity in relation to the INRG clinicopathological criteria are shown in Additional file 1: Table S1.

**Table 1:** Description of the vitronectin and nuclei morphometric measurements of control tissues and NB primary tumors

Parameter			Median	Mean	SD	Range	Q_3_
Normal control tissues other than liver (*n* = 12)							
IPP	Nuclei	Density	308.10	305	99.75	94.10–477.70	355
		%SA	7.30	7.90	3.35	4–14	8.80
	Inter. VN	%SA	1.57	3.92	5.70	0.42–17	3.67
	Terr. VN	%SA	0.04	0.07	0.07	0–0.20	0.15
Densito	VN ratio of pixels	Weak	0.04	1	1.50	0–4	1.75
		Moderate	2.25	10.95	13	0.06–35	22.90
		Strong	1	2	2.60	0–9	2.95
	H-score		20.45	46.40	52.51	0.16–163	82.50
Liver samples (*n* = 5)							
IPP	Nuclei	Density	616	1846.70	2384.30	472–6051.70	3767.80
		%SA	4	3.80	2.05	2–7	5.50
	Inter. VN	%SA	20	19	11.40	3–31	29.50
	Terr. VN	%SA	3	2.50	1.30	0.65–4	3.50
Densito	VN ratio of pixels	Weak	2.50	2.85	2.30	0.30–6	5.15
		Moderate	67	64.55	8.30	50–71	69.50
		Strong	12.75	13.50	2.70	10–17	16
	H-score		175.80	172.60	11.60	152.25–181.35	179.55
Primary tumors (*n* = 91)							
IPP	Nuclei	Density	560	1074.25	1455.80	95–6793	739
		%SA	11.75	11.70	6.30	0.35–29.70	15.50
	Inter. VN	%SA	9.35	12.25	10.95	0.02–40.97	18.20
	Terr. VN	%SA	0.71	2.95	4.45	0.01–20.70	4.50
Densito	VN ratio of pixels	Weak	0.80	1.60	2.40	0.01–14.65	2.10
		Moderate	35	36.10	24.70	0.05–88.10	56.75
		Strong	3.55	12.85	17.85	0.01–61.85	20.40
	H-score		110.91	112.85	78.55	1.07–257.60	183.80

Descriptors of vitronectin (VN) immunoreactivity and nuclei according to their morphometric measurements are shown. IPP: Image Pro-Plus; Densito: DensitoQuant; Inter.VN: Interterritorial VN; Terr.VN: Territorial VN. Density: number of objects/mm^2^; %SA: percentage of stained area. SD: Standard deviation; Q_3_: third quartile

### High territorial VN expression pattern is associated with poor prognostic factors

Mann-Whitney and Kruskal-Wallis tests demonstrated that strong VN intensity, territorial VN and high H-score were statistically associated with unfavorable prognostic factors. These VN features were present in tumors from patients with metastatic stage (excluding high H-score), uNB/pdNB histopathology, MNA, SCA profile, 11qD (excluding high H-score), high-risk pretreatment stratification group and high genetic instability. No significant statistical relationship was observed between VN quantity and ploidy or histopathology category. The quantity of cell nuclei was higher in samples from patients aged ≥18 months, metastatic stage, uNB/pdNB histopathology, MNA, high-risk pretreatment stratification and high genetic instability. *p*-values for the relationship between VN patterns and INRG pre-treatment risk classification are shown in Table 2.

**Table 2:** *p*-values and relationship between vitronectin and nuclei morphometric measurements and poor prognostic factors

Parameter			Age: ≥18 months	Stage: M	Hist.D: uNB/pdNB	*MYCN:* MNA	Gen. profile: SCA	11q: 11qD	Risk group: high-risk	Gen. Instab.:High
IPP	Nuclei	Dens.	–	0.005↑	0.007↑	0.001↑	–	–	0.001↑	0.003↑
		%SA	0.004↑	–	–	–	0.018↓	–	–	–
	Inter.VN	%SA	–	–	–	–	–	–	–	–
	Terr.VN	%SA	–	0.010↑	0.001↑*	0.024↑	0.001↑	0.035↑	0.008↑	0.000↑
Densito	VN ratio of pixels	Weak	–	–	–	–	–	–	–	–
		Mod.	–	–	–	–	–	–	–	–
		Strong	–	0.037↑	0.010↑*	0.011↑	0.002↑	0.021↑	0.004↑	0.010 ↑
	H-score		–	–	0.019↑	0.040↑	0.011↑	–	0.008↑	0.001↑

Only morphometric variables for vitronectin (VN) expression and nuclei having a statistically significant relationship with pre-treatment risk stratification factors are shown (*p*-value< 0.05). IPP: Image Pro-Plus; Densito: DensitoQuant; Dens. Density (number of objects/mm2); %SA: percentage of stained area; Inter. VN: Interterritorial VN; Terr.VN: Territorial VN; Mod.: moderate. M: metastatic; Hist.D: histopathologic differentiation; uNB: undifferentiated neuroblastoma; pdNB: poorly differentiated neuroblastoma; NOS was excluded from statistical analysis; Gen. Profile: genetic profile; SCA: segmental chromosomal aberration; MNA: *MYCN* amplified; 11qD: 11q deletion; Gen. Instab.: genetic instability. -: not statistically significant, ↑/↓: higher or lower median value for the poor-prognostic group(s). *There are statistically significant differences between pdNB and uNB, within Strong VN (*p*-value< 0.05) and %SA Territorial VN (*p*-value< 0.1) morphometric variables

### The highest territorial VN expression pattern is related to poor survival

**Fig. 2:**
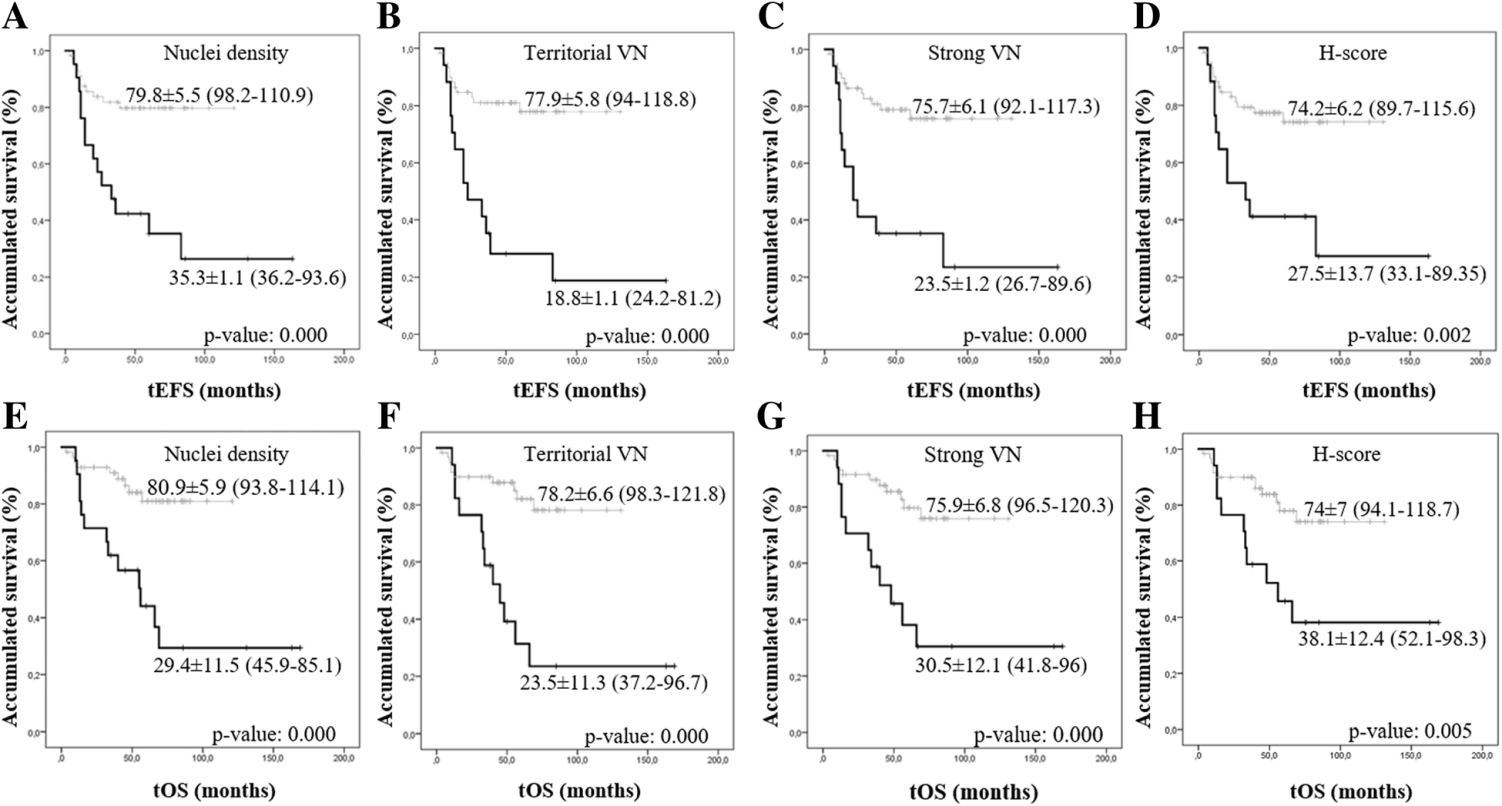
Kaplan–Meier graphs showing the EFS (**a**-**d**) or OS (**e**-**h**) depending on different variables. In all cases, the black and grey lines correspond to the group above and below the third quartile (Q_3_) used for dichotomization, respectively. Only morphometric variables for vitronectin (VN) quantity with a statistically significant relationship with event-free survival (EFS) and/or overall survival (OS) are shown (*p*-value< 0.05). *p*-values and 5-year survival rates are shown. **a** and **e** Nuclei density. **b** and **f** Territorial VN. **c** and **g** VN Strong ratio of pixels. **d** and **h** H-score

Samples with the highest VN levels and corresponding to strong staining intensity, territorial location and H-score ≥ Q_3_ were associated with poorer 5-year EFS and lower 5-year OS, compared to patients whose samples presented a low VN level (<Q_3_), (*p*-value< 0.05). Furthermore, samples with nuclei density ≥ Q_3_ were related to poorer 5-year EFS and lower 5-year OS (Fig. 2). To perform the multivariate survival analysis using Cox proportional hazards regression, we considered all INRG variables together with the VN morphometric variables considered as statistically significant by the log-rank test (Kaplan-Meier curves). This test showed that age ≥ 18 months, uNB/pdNB histopathology and 11qD remained significant predictors for EFS. OS was influenced by age ≥ 18 months, 11qD (*p*-value< 0.05), MNA and territorial VN expression (*p*-value< 0.1) (Table 3).

**Table 3:** Cox Regression of morphometric vitronectin variables and INRG prognostic factors

Variable	B	S.E	Wald	Exp (B) (95% CI)	*p*-value
EFS					
Age (≥18 month)	1.361	0.526	6.696	3.898 (1.39–10.92)	0.010
Hist.D (uNB/pdNB)	1.073	0.411	6.806	2.924 (1.30–6.54)	0.009
11q status (11qD)	0.883	0.424	4.332	2.418 (1.05–5.55)	0.037
OS					
Age (≥18 month)	1.320	0.603	4.797	3.745 (1.14–12.20)	0.029
11q status (11qD)	1.309	0.469	7.785	3.702 (1.47–9.28)	0.005
*MYCN (MNA)	0.857	0.472	3.296	2.357 (0.94–5.94)	0.069
*Terr. VN_Q_3_	0.852	0.480	3.154	2.344 (0.92–6)	0.076

Significant INRG prognostic parameters and morphometric vitronectin (VN) measurements predictive of poor outcome in neuroblastoma (NB) patients based on event-free survival (EFS) and overall survival (OS) with *p*-value < 0.05 and *p-value < 0.1. Hist.D: histopathologic differentiation; uNB: undifferentiated neuroblastoma; pdNB: poorly differentiated neuroblastoma; 11qD: 11q deletion; MNA: *MYCN* amplified; Terr.VN_Q_3_: Territorial vitronectin dichotomized at the third quartile. B: Beta coefficient; S.E: Standard Error; CI: Confidence interval. Coefficients Exp (B) > 1 indicate that high values of this parameter increase the probability of it being an independent poor prognostic factor

### In vitro and in vivo studies demonstrate VN expression by malignant neuroblasts

**Fig. 3:**
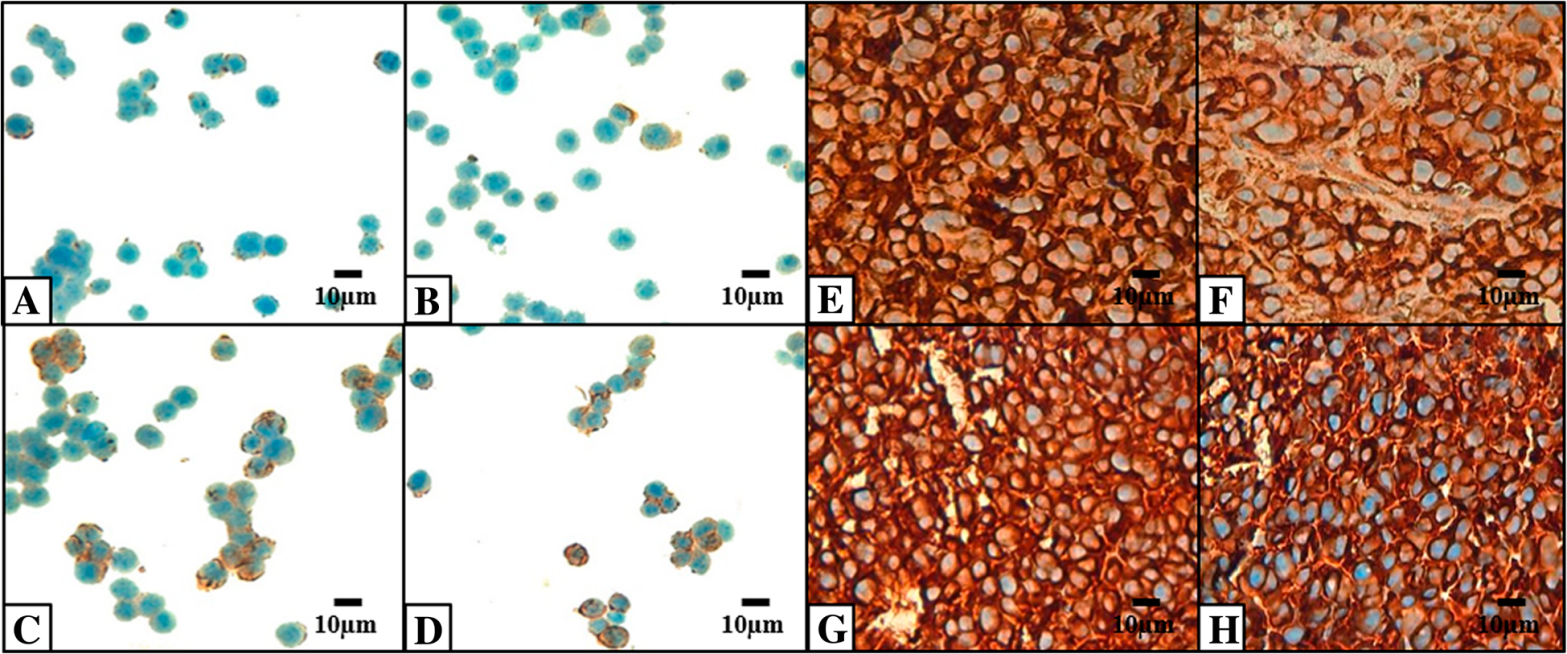
Examples of in vitro (**a-d**) and in vivo (**e**-**h**) vitronectin expression. Vitronectin (VN) expression in SK-N-BE (2) cells maintained in: **a** Complete medium. **b** Serum-free medium. VN expression in SH-SY5Y cells maintained in: **c** Complete medium. **d** Serum-free medium. VN expression in orthotopic neuroblastoma (NB) mice after SK-N-BE (2) injection. **e** RAG1^−/−^ VN^+/+^ mice. **f** RAG1^−/−^ VN^−/−^ mice. VN expression in orthotopic NB mice after SH-SY5Y injection. **g** RAG1^−/−^ VN^+/+^ mice. **h** RAG1^−/−^ VN^−/−^ mice

Regarding VN expression in malignant neuroblasts, we observed low amounts of cytoplasmic VN dot drops expression in around 50% of cells in both growth conditions (complete and serum-free media) of both cell lines (Fig. 3). Tumor development after SH-SY5Y and SK-N-BE (2) inoculation was observed in RAG1^−/−^ VN^+/+^ mice (SH-SY5Y = 7/10 and SK-N-BE (2) = 10/10) and RAG1^−/−^ VN^−/−^ mice (SH-SY5Y = 9/10 and SK-N-BE (2) = 9/10). We found no significant differences between tumor growth in control compared to experimental RAG1^−/−^ VN^−/−^ in any of the cell lines. Tumors displayed small and limited intra-adrenal, and large abdominal solid masses with a heterogeneous macroscopic appearance, as well as moderate infiltrative growth into surrounding tissues such as perirenal fat, pancreas and liver, both in animal models and in NB cell lines. Regarding histopathology, solid tumors from the SH-SY5Y cell line presented 30–90% undifferentiated neuroblastic cells, non-evident nucleolus, high mitosis-karyorrhexis index (IMK) and between 5 and 50% necrosis. With respect to the samples derived from cell line SK-N-BE (2), these were characterized by being uNB with a mean of 40–70% neuroblasts with evident nucleolus, high IMK, and 5–50% necrosis. Highly dense cytoplasmic VN staining was similar in control and experimental mice derived from both cell lines (Fig. 3).

## Discussion

VN is an attachment glycoprotein that directs cell migration, progression, adhesion and differentiation in many biological and pathological processes [13, 15, 30, 31]. By introducing advanced morphometric methodology, we have been able to demonstrate and robustly quantify VN as an important ECM component in aggressive NB synthetized by undifferentiated neuroblasts. We have found two VN expression patterns which reflect the secretion time and role of this glycoprotein that has typically been described for ECM constituents in cartilage: the territorial VN pattern, where VN is synthetized and recently incorporated to the newly formed matrix facilitating mechanical stresses of tumor cells, and the interterritorial VN pattern, where VN has been integrated some time ago to the matrix contributing to metabolic and biomechanical properties of several tumor tissue elements.

Some contradictory studies of VN expression in NB have been described [12, 27, 32]. Gladson et al. 1997, showed VN expression in differentiating neuroblastic tumors and noted its attachment role in retinoic-acid differentiated neuroblastic cells stimulating in vivo differentiation [12]. Later NB studies detected an overexpression of α_v_β_3_ integrin and uPAR, both of which are important VN ligands, in high-risk NB [27, 32]. From a larger cohort of neuroblastic tumors, our immunohistochemical results revealed VN expression in NB and GNB, with a high territorial VN expression in undifferentiated neuroblasts. Our findings are in agreement with the most recent studies mentioned above and the differences from the earlier study [12] could be explained by cohort size. Using digital pathology techniques, we were able to accurately provide a connection between intensity and location. These techniques assure the standardization of all measurements and minimize inter-observer differences [33, 34] and can easily be reproduced in future studies. We are currently carrying out topological analysis on the histopathological images to evaluate non-cellular VN distribution features related to the tumor microenvironment. Our topological approach will capture different aspects of the VN distribution to improve the classification of biopsy samples from NB samples.

The in vitro and vivo preliminary results in the present study mainly focused on human NB, were to emphasize the VN secretion by both NB cell lines and highlight the tumor and/or host microenvironment influence in their VN synthesis. We found that in vitro NB cells expressed a low quantity of VN as a small cytoplasmic dot drops pattern and that the in vivo experiments revealed a high amount of VN as a dense cytoplasmic pattern with no differences in VN staining pattern or tumor growth rate between RAG1^−/−^ VN^+/+^ and RAG1^−/−^ VN^−/−^ mice. The increased VN secretion by tumor cells, which produces mechanical stress, generates an initial stiff matrix and results in disrupted cell-cell and cell-ECM interactions, promoting tumor proliferation in both mice strains, as described in ovarian cancer, through the breakdown of  VN bonds to improve metastasis [35]*.* We confirmed that: a) Fetal bovine serum (FBS) does not contribute to a higher amount of VN in tumor cells as reported by Gladson et al. [36] and b) the importance of VN in NB tumor growth due to its high presence in vivo. These findings reinforce the previously described VN role in improving the migratory ability of tumor cells in NB and suggest that in vivo tumor cells produce VN to achieve greater migratory capacity. Furthermore, given the importance of VN in breast cancer [37], we are developing experiments to clarify the role of VN in vivo in NB tumor growth. Serial tumor passages will allow VN secretion by neuroblasts to be modified in relation to a host microenvironment, affecting tumor growth and genetic instability, as occurs in hormone-dependent tumors [38]. Therefore, although xenotranplanted neuroblastic cells continue to synthetize VN, the absence of liver VN in the host would have a subtle role in the secretory ability of malignant neuroblasts, but some long-term influence on cell proliferation and tumor aggressiveness.

We stress that a high territorial VN expression pattern could contribute to tumor cell adhesion, thus promoting invasion and metastasis in NB, suggesting that VN or its ligands could be used as targets when developing therapeutic strategies for modulating the relationship between tumor cells and the ECM. In fact, the highest presence of territorial VN protein in the present cohort is related to unfavorable independent prognostic INRG variables. The high presence of territorial VN staining in tumors from patients with metastasic stage and unfavorable histology, would lead to higher migration ability of tumor cells by anchorage to fibers and proteoglycans, as well as disrupted cell adhesion and spreading via interactions with specific α_ν_β_3_ and α_ν_β_5_ integrins, uPAR and PAI-1 in a stiff matrix. In the case of tumors with genetic instability, a huge presence of territorial VN would generate mechanical alterations in ECM, which would be transmitted to the nuclear matrix and would modulate the response of intracellular signals activating genetic and epigenetic mechanisms of instability in these tumors [39].

The main therapeutic goal would be to focus on decreasing VN expression or to inhibit its joining with α_v_β_3_ integrin, uPAR or PAI-1, thus depriving the tumor cells of the mechanical forces necessary to create the appropriate environment for invasion [40–42]. Via its ligands, VN has a role in biochemical cell-ECM pathways which could be used as therapeutic targets; however, no drug against cell-ECM interactions has yet been approved, although some trials are ongoing as described below.

α_v_β_3_ and α_v_β_5_ integrins bind to the arginine-glycine-aspartate (RGD) VN sequence and are key factors in angiogenesis. It has been observed that VN activates vascular endothelial growth factor receptor 2 (VEGFR-2) via α_v_β_3_, thus contributing to angiogenesis [43]. As anti-α_v_β_3_ integrin targeted drugs: Vitaxin, Intetumubab (CNTO 95) and 17E6 (EMD 525797) are in different phases of clinical trials [44], also in vitro melanoma research has displayed metastasis retardation using IH1062, an α_v_β_3_ integrin inhibitor that blocks the binding of this integrin to VN [45].

uPAR is a protease that binds to VN in the RGD motif or N-terminal somatomedin B (SMB) domain. It has been observed that this union initiates integrin pathways that promoting migration [46, 47]. In addition, when uPAR binds to urokinase plasminogen activator (uPA), it causes the cleaving of plasminogen to produce plasmin that mediates the degradation of the ECM [48]. An in vivo study into a monoclonal anti-uPAR antibody (ATN-658) reported that this antibody inhibits tumor cell proliferation in prostate cancer [49].

Finally, PAI-1 is a protease inhibitor of the serpins family that binds to the SMB domain of VN where its main function is uPA inhibition. Thus, PAI-1 participates in the uPA/uPAR proteolytic cascade, as well as interfering in the binding of uPAR and α_v_ integrins family in the SMB domain and RGD motif to VN, respectively [50]. In fact, an in vivo study of NB using PAI-1 deficient mice showed a reduction in tumor size [51]. In vitro and in vivo studies on the inhibition of PAI-1 using compounds such as TM5441, TM5275 and SK-216 have demonstrated toxic effects in cancer cells [52, 53].

## Conclusions

In conclusion, NB samples of patients with poor prognostic factors are characterized by the highest territorial VN expression pattern. Our findings suggest the importance of extensive studies on VN as a possible target for inhibiting interactions in NB.

## Additional files

Additional file 1: **Table S1.** Descriptors and median values of vitronectin and nuclei morphometric variables in the present cohort. (DOCX 19 kb)

Additional file 2: **Table S2.** Description of the image analysis process. (DOCX 15 kb)

Additional file 3 **Figure S1.** Examples of how these applications work in vitronectin samples. A. Liver sample image immunostained for vitronectin (VN) without segmentation. B. Image of liver control sample segmentation with the DensitoQuant module (Pannoramic viewer software). C. Image of liver control sample segmentation with Image Pro-Plus software. D. Primary neuroblastoma (NB) sample immunostained for VN without segmentation. E. Image of NB sample segmentation with the DensitoQuant module (Pannoramic viewer software). F. Image of NB sample segmentation with Image Pro-Plus software. (ZIP 8306 kb)

Additional file 4: **Table S3.** Growth conditions of NB human cell lines. (DOCX 14 kb)

## Abbreviations

%SA: Percentage of stained area
11qD: 11q deleted
11qND: 11q non-deleted
DensitoQ: DensitoQuant
dNB: Differentiating neuroblastoma
ECM: Extracellular matrix
EFS: Event-free survival
GNB: Ganglioneuroblastoma
INRG: International Neuroblastoma Risk Group
IPP: Image Pro-Plus
MNA: *MYCN* amplified
MNNA: *MYCN* non-amplified
NB: Neuroblastoma
NCAs: Numerical chromosomal aberrations
OS: Overall survival
PAI-1: Plasminogen activator inhibitor-1
pdNB: Poorly differentiated neuroblastoma
Q_3_: Third quartile
RGD: Arginine-glycine-aspartate motif
SCAs: Segmental chromosomal aberrations
SMB: Somatomedin B domain
TMAs: Tissue microarrays
uNB: Undifferentiated neuroblastoma
uPA: Urokinase plasminogen activator
uPAR: Urokinase plasminogen activator receptor
VN: Vitronectin
