# Supplementary material for: Vitronectin as a molecular player of the tumor microenvironment in neuroblastoma
Source: BMC Cancer. 2019 May 22;19:479. doi: 10.1186/s12885-019-5693-2 (PMC6532218; doi:10.1186/s12885-019-5693-2)
Supplement: Supplementary file 1 — Table S1. Descriptors and median values of vitronectin and nuclei morphometric variables in the present cohort. (DOCX 19 kb) [file 12885_2019_5693_MOESM1_ESM.docx]

**Additional file 1: Table S1.** Descriptors and median values of vitronectin and nuclei morphometric variables in the present cohort.

| **Variable and category** | | **Nº**  **cases** | **Nuclei** | | **Inter.**  **VN** | **Terr. VN** | **VN ratio of pixels** | | | **H-score** |
| --- | --- | --- | --- | --- | --- | --- | --- | --- | --- | --- |
|  |  |  | Density | %SA | %SA | %SA | Weak | Mod. | Strong |  |
| Age | <18months | 51 | 534.15 | 12.95 | 10 | 0.66 | 0.70 | 34 | 3.20 | 95.40 |
|  | ≥18months | 40 | 614.20 | 9.45 | 7.95 | 0.85 | 0.82 | 37.80 | 3.90 | 131.30 |
| Stage | L1,L2,MS | 65 | 527.25 | 12 | 9.90 | 0.55 | 0.82 | 34.30 | 2.50 | 80.80 |
|  | M | 23 | 669.40 | 9.90 | 9.10 | 2.65 | 0.60 | 37.90 | 13.45 | 143.40 |
| Hist. C. | GNB | 9 | 219 | 6.50 | 2 | 0.50 | 1 | 44.25 | 4 | 80.10 |
|  | NB | 82 | 570.60 | 12.20 | 9.90 | 0.75 | 0.75 | 34.60 | 4.39 | 111.30 |
| Hist.D. | dNB | 10 | 456.25 | 13.90 | 5.95 | 0.20 | 0.85 | 30.25 | 2 | 71.25 |
|  | pdNB | 57 | 563.94 | 12.20 | 9.90 | 0.80 | 0.80 | 36.40 | 3.55 | 110.70 |
|  | uNB | 13 | 1567 | 8.60 | 11.80 | 6.30 | 0.60 | 34.30 | 24.60 | 198 |
| MYCN | MNNA | 71 | 534.15 | 12.20 | 11.80 | 0.50 | 0.80 | 34.30 | 2.25 | 97.30 |
|  | MNA | 19 | 2173.90 | 8.60 | 9 | 2.30 | 0.60 | 37.70 | 17.12 | 156.80 |
| 11q | ND | 65 | 563.95 | 12.50 | 10 | 0.60 | 0.95 | 34.95 | 2.85 | 110.90 |
|  | D | 19 | 603.40 | 9.60 | 13.10 | 2.90 | 0.75 | 38.35 | 16 | 143.75 |
| Ploidy | Hiperp. | 48 | 570.60 | 12.10 | 9.95 | 1.35 | 0.60 | 38 | 5.25 | 112.95 |
|  | Dip+tetrap | 11 | 739 | 9.80 | 10.50 | 0.90 | 0.95 | 36.35 | 5.60 | 137 |
| Gen. profile | NCA | 26 | 551.40 | 14.10 | 5.80 | 0.30 | 1.10 | 21.60 | 1.60 | 69.80 |
|  | SCA | 57 | 600.60 | 11.60 | 11.80 | 1.30 | 0.80 | 37.90 | 7.10 | 143.40 |
| Risk group | Non-HR | 64 | 530.70 | 12.60 | 9.10 | 0.50 | 0.80 | 32.30 | 2.20 | 81.60 |
|  | HR | 27 | 1120 | 9.40 | 9.90 | 2.30 | 0.80 | 38.37 | 13.45 | 163.70 |
| Gen.  Instab. | Low | 46 | 538.60 | 13.60 | 9.20 | 0.43 | 1 | 27 | 1.70 | 75.70 |
|  | High | 26 | 793 | 9.10 | 10.85 | 2.87 | 0.70 | 38.36 | 16.57 | 169.87 |

Density: number of objects/mm^2^; Inter. VN: Interterritorial VN; Terr.VN: Territorial VN; %SA: percentage of stained area. L1 and L2: localized and MS: special metastasic; M: metastatic; Hist. C.: histopathologic category; GNB: ganglioneuroblastoma; NB: neuroblastoma; Hist. D: histopathologic differentiation; dNB: differentiating neuroblastoma; pdNB: poorly differentiated neuroblastoma; uNB: undifferentiated neuroblastoma; NOS was excluded from statistical analysis; MNNA: *MYCN* non-amplified; MNA: *MYCN* amplified; ND: non deletion; D: deletion; Gen. Profile: genetic profile; NCA: numerical chromosomal aberration; SCA: segmental chromosomal aberration; Hiperp.: Hiperploid; Dip.: diploid; Tetrap.:tetraploid; HR: High-risk; Gen. Instab.:genetic instability.
