# Supplementary material for: Vitronectin as a molecular player of the tumor microenvironment in neuroblastoma
Source: BMC Cancer. 2019 May 22;19:479. doi: 10.1186/s12885-019-5693-2 (PMC6532218; doi:10.1186/s12885-019-5693-2)
Supplement: Supplementary file 2 — Table S2. Description of the image analysis process. (DOCX 15 kb) [file 12885_2019_5693_MOESM2_ESM.docx]

**Additional file 2: Table S2.** Description of the image analysis process.

| **Element** | **Material** | **Software** | **Algorithm segmentation** |
| --- | --- | --- | --- |
| VN | -Individual images  -JPEG format, quality 80  -RGB colour model | Image Pro-Plus (IPP) software v.6.0 (Media Cybernetics Inc., Silver Spring, MD, USA) | Customized macro:  -Image pre-processing: contrast enhancement to better distinguish between blue and brown hues, in the following values: 50, 70, and 1 and for the high intensity VN cases: 70,70,1.  -Morphological filters: Erode/Dilate and the enhancement HiGauss filter. In this step, an optimization of the appearance of the image without altering the true immunoreactivity of the cases was got to facilitate the segmentation process.  -RGB channel segmentation:  • The values for nuclei segmentation were R: 0-169, G: 0-214 and B: 82-255 (R: 0-202, G: 0-214 and B: 43-255 for high intensity VN cases). To a proper nuclei separation we used Autosplit and Watershed, followed by a restriction area >10μm^2^.  • The color ranges for interterritorial VN were R:166-255, G:4-193 and B:0-135 (R:166-255, G:39-123 and B:0-135 for high intensity VN cases) and for territorial VN R:0-90, G:0-90 and B:0-90 (R:0-136, G:0-90 and B:0-90 for high intensity VN cases). A restriction area > 5μm^2^ was used in both cases. |
|  | -Whole-digitized slide  (identification of  cylinders corresponding  to the different samples)  -TIFF format  -RGB color model | Pannoramic viewer software1.15 (3DHISTECH Ltd., Budapest, Hungary) | DensitoQuant module:  -Color adjustments: Blue detection: 0.8 and brown tolerance: 1. (In high VN intensity images the brown tolerance changes to 1.8).  -The score levels were: 6.41, 15.13, and 44.28. (In case of high VN intensity images, the last score level 44.28 changes to 56.14). |

VN: Vitronectin; JPEG: joint photographic expert group format; RGB: R=red, G=green and B=blue color model; TIFF: tagged image file format.
