# Supplementary material for: Vitronectin as a molecular player of the tumor microenvironment in neuroblastoma
Source: BMC Cancer. 2019 May 22;19:479. doi: 10.1186/s12885-019-5693-2 (PMC6532218; doi:10.1186/s12885-019-5693-2)
Supplement: Supplementary file 3 — Figure S1. Examples of how these applications work in vitronectin samples. A. Liver sample image immunostained for vitronectin (VN) without segmentation. B. Image of liver control sample segmentation with the DensitoQuant module (Pannoramic viewer software). C. Image of liver control sample segmentation with Image Pro-Plus software. D. Primary neuroblastoma (NB) sample immunostained for VN without segmentation. E. Image of NB sample segmentation with the DensitoQuant module (Pannoramic viewer software). F. Image of NB sample segmentation with Image Pro-Plus software. (ZIP 8306 kb) [file 12885_2019_5693_MOESM3_ESM.zip › Additional file 3.docx]

**Additional file 3: Figure S1.** Examples of how these applications work in vitronectin samples. **A**. Liver sample image immunostained for vitronectin (VN) without segmentation. **B**. Image of liver control sample segmentation with the DensitoQuant module (Pannoramic viewer software). **C**. Image of liver control sample segmentation with Image Pro-Plus software. **D.** Primary neuroblastoma (NB) sample immunostained for VN without segmentation. **E.** Image of NB sample segmentation with the DensitoQuant module (Pannoramic viewer software). **F.** Image of NB sample segmentation with Image Pro-Plus software. Color coding of VN analysis: in DensitoQuant segmented image, blue=negative, yellow=weak, orange= moderate and red=strong; in Image Pro-Plus segmented image, green=nuclei, brown= weak to moderate (interterritorial VN) and red=strong (territorial VN).
