# Supplementary material for: Vitronectin as a molecular player of the tumor microenvironment in neuroblastoma
Source: BMC Cancer. 2019 May 22;19:479. doi: 10.1186/s12885-019-5693-2 (PMC6532218; doi:10.1186/s12885-019-5693-2)
Supplement: Supplementary file 4 — Table S3. Growth conditions of NB human cell lines. (DOCX 14 kb) [file 12885_2019_5693_MOESM4_ESM.docx]

**Additional file 4: Table S3.** Growth conditions of NB human cell lines.

| **Complete medium** | **Serum-free medium** |
| --- | --- |
| Iscove's Modified Dulbecco's Medium (IMDM; Gibco; Thermo Fisher Scientific Inc., Waltham, MA, USA). | Iscove's Modified Dulbecco's Medium (IMDM; Gibco; Thermo Fisher Scientific Inc., Waltham, MA, USA). |
| 10% Fetal bovine serum (FBS; Gibco; Thermo Fisher Scientific Inc.). | F-12 (F-12 Nutrient Mix, GlutaMAX™; Gibco; Thermo Fisher Scientific) |
| Insulin-transferrin-selenium (ITS; Gibco; Thermo Fisher Scientific, Inc.). | Insulin-transferrin-selenium (ITS; Gibco; Thermo Fisher Scientific, Inc.). |
| 100 U/mL penicillin/100 μg/mL streptomycin (Gibco; Thermo Fisher Scientific Inc.). | 100 U/mL penicillin/100 μg/mL streptomycin (Gibco; Thermo Fisher Scientific Inc.). |
| Plasmocin (Ibian Technologies, S.L., Zaragoza, Spain) to prevent mycoplasma contamination. | Plasmocin (Ibian Technologies, S.L., Zaragoza, Spain) to prevent mycoplasma contamination. |
